# First Characterization and Zoonotic Potential Evaluation of *Giardia duodenalis* in Ferrets in China

**DOI:** 10.1155/tbed/3087035

**Published:** 2025-05-29

**Authors:** Heng Yang, Xiaocen Wang, Xu Zhang, Yanhui Yu, Chaofan Li, Hongyu Wang, Xuewei Fan, Pengtao Gong, Nan Zhang, Xin Li, Jianhua Li

**Affiliations:** ^1^State Key Laboratory for Diagnosis and Treatment of Severe Zoonotic Infectious Diseases, Key Laboratory for Zoonosis Research of the Ministry of Education, Institute of Zoonosis, and College of Veterinary Medicine, Jilin University, Changchun, Jilin, China; ^2^Second Affiliated Hospital, Jilin University, Changchun, Jilin, China; ^3^Department of Animal Science and Technology, Heilongjiang Agricultural Economy Vocational College, Mudanjiang, Heilongjiang, China

**Keywords:** epidemiology, ferret, *G. duodenalis*, pathogenicity, whole genome sequencing

## Abstract

*Giardia duodenalis* is a protozoan parasite that causes an important zoonosis in humans and a number of mammals, but has been poorly reported in ferrets. In the present study, we obtained a *G. duodenalis* isolate from a pet ferret and cultured it *in vitro*. The morphological character was consistent with *G. duodenalis*, being inverted pear-shaped with four pairs of flagella. The trophozoites measured 15.0 ± 1.2 μm (*n* = 50) in length and 7.5 ± 0.8 μm (*n* = 50) in width. The genome of the new isolate was 12.1 Mbp, which was relatively larger with a high guanine–cytosine (GC) content compared to *G. duodenalis* isolates from other hosts. 720 variant genes were identified, suggesting that the isolate might have evolved unique genetic features, potentially reflecting differences in its adaptive or pathogenic capabilities. The pathogenicity experiments revealed that the infection caused significant duodenal lesions, characterized by villous atrophy and breakage. Additionally, varying degrees of pathological changes were observed in other intestinal segments, and the infected animals exhibited a reduced rate of weight gain compared to the control group. In addition, molecular identification showed that the isolate belonged to zoonotic assemblage A at all three loci (*tpi*, *bg*, and *gdh*). Furthermore, the infection rate among 111 ferret fecal samples was 14.41%, with assemblage A as the dominant genotype. The sequence of *G. duodenalis* obtained from the genome of ferret feces in this study was more closely related to the Japanese isolates in East Asia in terms of phylogenetics and more distantly related to the German isolates in Europe. In conclusion, these findings suggested that *G. duodenalis* in ferrets exhibited high zoonotic potential, and the genomic and epidemiological data provided an important theoretical basis for future studies on the transmission and evolution of *G. duodenalis*.

## 1. Introduction

*Giardia duodenalis* is a flagellated protozoan parasite [1]. It commonly parasitizes the gastrointestinal tract of humans as well as a wide range of mammals to cause giardiasis, which is characterized by diarrhea, weight loss, and malabsorption [2]. *G. duodenalis* also harms the growth, development, and cognitive function of infected children [3]. *G. duodenalis* has a global distribution and has been implicated in multiple outbreaks worldwide [4]. Approximately 280 million symptomatic human cases are reported annually worldwide, with an even more pronounced impact in developing countries, where infection rate may reach 20% to 30% [5]. Giardiasis leads to substantial economic losses annually, posing a severe threat to global public health [6]. It has been included in the World Health Organization (WHO) Neglected Diseases Initiative since 2004 [7]. Giardiasis is also endemic in livestock and companion animals, thereby exerting a significant impact on public and veterinary health [8].

*G. duodenalis* represents a complex species comprising eight distinct assemblages with varying host specificities, classified as assemblages A to H [9]. Assemblages A and B are capable of infecting both humans and a variety of mammals, thereby demonstrating a broad host range with significant zoonotic potential. In contrast, assemblage C–H exhibits host specificity for certain animals species and presents a lower risk of human infection [10, 11]. Assemblage A can be further subdivided into three well-defined phylogenetic clusters, subassemblages AI, AII, and AIII, each exhibiting distinct host selection. Subassemblage AI predominantly occurs in animals such as cattle, sheep, pigs, cats, and dogs, and it has also been documented in human through sporadic cases [12]. Conversely, subassemblage AII is primarily associated with human infections, and subassemblage AIII is primarily detected in wild ruminant species [13]. Whole-genome data are crucial in establishing phylogenetic relationships and identifying genotype-specific markers for epidemiological research [14]. This provides a novel approach to gain insights into genetic diversity at the subspecies level.

*G. duodenalis* has a relatively simple life cycle, comprising two stages: invasive trophozoites and infectious cysts [15]. Transmission of *G. duodenalis* occurs via multiple routes, including direct person-to-person contact and indirect transmission through contaminated water or food [16]. The infection in household pets has notably garnered increasing attention. *G. duodenalis* has been found in companion animals (e.g., cats and dogs) in China. In recent years, the popularity of ferrets as a household pet has surged due to their friendly nature, small size, and relative ease of breeding and care. Globally, epidemiological data on *G. duodenalis* infection in ferrets remain scarce. Currently, the prevalence of *G. duodenalis* in ferrets has only been investigated in a few countries such as the UK and Italy [17, 18] and molecular epidemiological data from ferrets in China have not been reported.

To explore the fundamental characteristics of *G. duodenalis* in ferrets, a systematic laboratory investigation was conducted. This investigation encompassed pathogen isolation and identification, whole-genome resequencing, infection experiments, and epidemiological studies, providing a comprehensive assessment of the current epidemiological status of *G. duodenalis* in ferrets. This study enhanced our understanding of the evolutionary relationship within *G. duodenalis*, thereby establishing a scientific foundation for the prevention of emerging *Giardia* species.

## 2. Materials and Methods

### 2.1. Purification of *G. duodenalis* Cysts

In this study, the pet ferret (8-month-old, male) exhibited clinical signs including depression, yellow and watery diarrhea and loss of appetite. The animal had no history of antiparasitic treatment and was housed indoors. Fresh feces were collected and identified by polymerase chain reaction (PCR), which confirmed the diagnosis of *G. duodenalis* infection. In addition, the pathogen (trophozoites and cysts) could be observed under optical microscopy. Subsequently, the positive fecal sample (15 g) was mixed with 10 mL of sterile water, then the mixture was filtered through a 60-mesh screen, the filtrate was separated by centrifugation at 3000 rpm for 10 min, and the supernatant was discarded. An equal amount of 33% zinc sulfate solution (Z820749, Macklin, China) was added to the precipitate, which was mixed and then centrifuged at 2000 rpm for 15 min, after which the supernatant was collected. The suspension was diluted by adding four times the volume of sterile water and centrifuged at 3000 rpm for 10 min. The supernatant was discarded, and the precipitate was resuspended in sterile water. Optical microscopic observation of *G. duodenalis* cysts after staining with Lugol solution. Finally, the solution was diluted in sterile water and stored at 4°C until used to infect gerbils.

### 2.2. Retrieval of New Isolate

The isolation of *G. duodenalis* trophozoites was performed as described previously with modifications [19]. Briefly, each 4-week-old female gerbil was orally gavaged with 1 × 10^4^ of the above purified cysts. Following infection, cysts was detected in the feces at 3 days post-infection (dpi). Gerbils developed clinical signs, including depression, loss of appetite, and soft stool. After euthanasia, the duodenums were sliced into 0.1 cm segments, placed in centrifuge tubes containing precooled phosphate-buffered saline (PBS) (containing 2% penicillin–streptomycin (E607011, Sangon Biotech, China), and pipetted repeatedly (Supporting Information Video S1). The sample was briefly centrifuged at 2000 rpm for 1 min to remove large fragments, and the supernatant was transferred to a new tube. The tube were centrifuged at 2500 rpm for 5 min, the supernatant discarded, and the precipitate was resuspended in trypticase-yeast extract-iron-serum 33 (TYI-S-33) medium (containing 2% penicillin–streptomycin), then incubated at 37°C for 30 min. Then replaced the medium (containing 2% penicillin–streptomycin) and incubated again at 37°C for 30 min. After 3–5 repetitions, the medium was replaced with TYI-S-33 medium (containing 1% penicillin–streptomycin), and incubated at 37°C. Finally, the isolated *G. duodenalis* trophozoites were cryopreservation in fetal bovine serum containing 10% dimethyl sulfoxide at –80°C for further morphological observation, staining and whole genome resequencing.

## 3. *G. duodenalis* Trophozoites Cultivation and Infection Experiment

The isolated *G. duodenalis* trophozoites were grown for 48 h in TYI-S-33 medium. Trophozoites were incubated on ice and then harvested by centrifuging at 3000 rpm for 10 min at 4°C. Following three times washing with sterile PBS, the trophozoites were counted. In the infection experiments, the gerbils were divided into seven groups, with five gerbils in each group. Animals received six doses (5 × 10^4^, 1 × 10^5^, 5 × 10^5^, 1 × 10^6^, 5 × 10^6^, or 1 × 10^7^) of trophozoites by oral gavage. The gerbils in the control group received PBS in the same manner. Cyst shedding and body weight were monitored daily for all animals, and pathological symptoms (if any) were recorded. In addition, fresh fecal samples were weighed immediately after collection (wet weight, ww) and dried at 60°C for 48 h before being weighed (dry weight, dw). The fecal moisture content represented the degree of intestinal health and was calculated as follows: Fecal moisture content (%) = (ww – dw)/ww × 100%.

### 3.1. Hematoxylin and Eosin (H&E) Staining

After gerbils were sacrificed, a 2 cm section of duodenum was removed, fixed in 10% formalin, embedded in paraffin, and then H&E staining was performed to assess pathological injury.

### 3.2. Scanning Electron Microscopy

The isolated *G. duodenalis* trophozoites were grown for 48 h in TYI-S-33 medium. The trophozoites were obtained by centrifuging and washed three times with PBS before they were fixed in a 2.5% glutaraldehyde solution (G5882, Sigma Aldrich, USA). The samples of *G. duodenalis* trophozoites were then sequentially dehydrated with a hierarchical ethanol series, freeze-dried, and gold sprayed, after which we observed the morphology of the trophozoites using a Hitachi S-3400N microscope (Hitachi, Tokyo, Japan)

### 3.3. Whole-Genome Resequencing

Genomic DNA was extracted from the isolated *G. duodenalis* trophozoites using a Blood & Tissue Genomic DNA Extraction Kit (TIANGEN, Beijing, China). These results were stored in a FASTQ (referred to as fq) format file, which contained sequence information of the reads and their corresponding sequencing quality information. Visual assessment of sequencing data quality of samples using FastQC (https://www.bioinformatics.babraham.ac.uk/projects/fastqc/). SNPs, and INDELs were identified utilizing the HaplotypeCaller algorithm, implemented in the Genome Analysis Toolkit. The reference genomes were obtained from *Giardia* DB, which corresponds to the *Giardia* WB strain. Raw DNA sequence reads from Illumina HiSeq were deposited at the NCBI Sequence Read Archive (SRA) under accession numbers SRR26945869. Whole-genome resequencing was performed by Shanghai Sangon Biotechnology Co., Ltd. (Shanghai, China).

### 3.4. Fecal Collection and DNA Extraction

To investigate the prevalence of *G. duodenalis* in ferrets, a total of 111 fresh ferret stool samples were collected between October 2022 and July 2023 from farms (*n* = 100) in Shandong province and from pet shops (*n* = 11) in Jilin province. Samples were labeled with provincial abbreviations and numbers (the samples in Jilin province, JL1−11; the samples in Shandong province, SD 1–100). All fecal samples were stored at –20°C prior for DNA extraction. Approximately 0.3 g of each fecal specimen was transferred to a 5 ml sterile tube, 2 ml of sterile water was added, and the tube was then thoroughly shaken with a vortex to homogenize the mixture. According to the manufacturer's instructions, genomic DNA was extracted from each fecal sample using the Fecal DNA Rapid Extraction Kit (TIANGEN, Beijing, China). The purified DNA samples were stored at –20°C until PCR analysis.

### 3.5. PCR Amplification

The presence of *G. duodenalis* in the fecal samples was detected via nested PCR for the *β*-giardin (*bg*), glutamate dehydrogenase (*gdh*), and triosephosphate isomerase (*tpi*) gene loci. The primer sequences and PCR conditions used were previously described (Supporting Information Table S1) [20]. The secondary PCR products were visualized by electrophoresis in 1% agarose gel containing ethidium bromide alternatives (D1210, APPLYGEN, China).

### 3.6. Sequence Analysis and Phylogenetic Analysis

The positive samples from the second round of PCR gel electrophoresis were sequenced and analyzed using the BLAST program (http://www.ncbi.nlm.nih.gov/BLAST/). The assemblages of *G. duodenalis* were determined using ClustalX 2.0.11 (http://clustal.org) against reference sequences downloaded from GenBank for each locus. Phylogenetic analysis of *G. duodenalis* was performed using MEGA X software. All nucleotide sequences obtained in this study have been deposited in GenBank under accession numbers PP578217-PP578238.

### 3.7. Statistical Analysis

Statistical analysis was performed using SPSS 26.0 statistical analysis software. Differences were considered statistically significant when *p* < 0.05. Infection rate and 95% confidence intervals (CIs) were also determined.

## 4. Results

### 4.1. Isolation and Identification of the Ferret Strain of *G. duodenalis*

**Figure 1:**
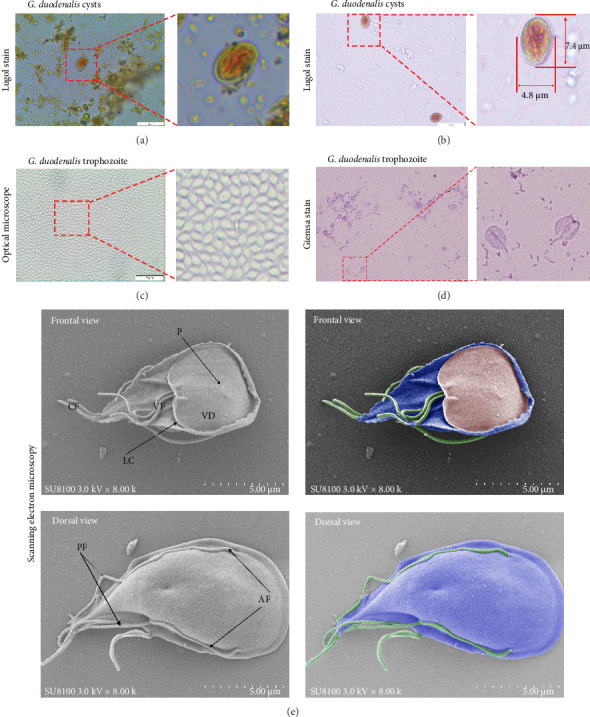
Isolation and characterisation of isolate of *G. duodenalis* in ferret. (A) *G. duodenalis* cysts were observed in ferret fecal samples after staining with Lugol's iodine. (B) Purified *G. duodenalis* cysts. (C) Trophozoites of new isolate of *G. duodenalis* cultured *in vitro*. (D) *G. duodenalis* trophozoites were observed after staining with Giemsa. (E) Scanning electron micrographs of *G. duodenalis* trophozoites. The ventral disc (VD) and the four pairs of flagella, the anterior flagella (AF), posterior–lateral flagella (PF), ventral flagella (VF), and caudal flagella (CF) were observed. Note its lateral crest (LC) and protrusion (P).

**Figure 2:**
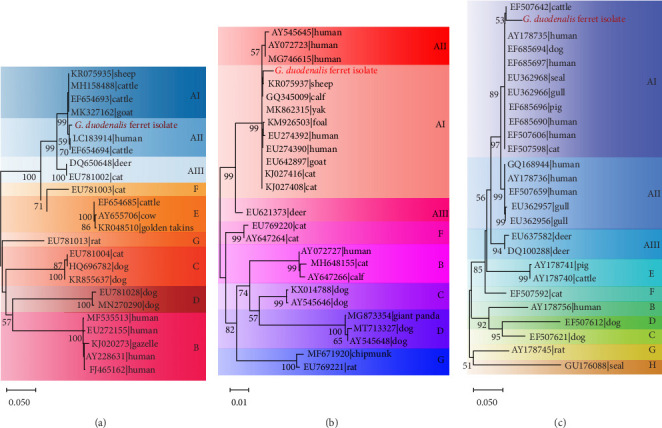
The phylogenetic tree analysis based on *tpi*,*bg*,and *gdh*. (A–C) Phylogenetic relationships of the sequences between *G. duodenalis* isolated in this study with those previously reported based on the nucleotide sequence at the three locus (*tpi* (A), *bg* (B), *gdh* (C)) and using a neighbor-joining Kimura 2-parameter method. Bootstrap values > 50% are shown.

A fecal sample exhibiting diarrhea was obtained from a pet ferret, and cysts were observed via Lugol's iodine staining. The cysts of the isolate were oval in shape with identifiable internal structures like the nucleus and axostyle, matching the morphology and size of *G. duodenalis* (Figure 1A, B). We successfully isolated *G. duodenalis* from the gerbil model *in vivo* and subsequently cultured *in vitro* using TYI-S-33 medium under anaerobic conditions at 37°C (Figure 1C). Notably, the trophozoites displayed a distinctive smile-like feature (Figure 1D), and scanning electron microscopy further revealed an inverted pear shape with a raised dorsal surface and four pairs of flagella, along with a clear ventral disc and protrusion. The trophozoites measured 15 ± 1.2 μm (*n* = 50) μm in length and 7.5 ± 0.8 μm (*n* = 50) (Figure 1E). Next, sequence analysis revealed the isolated *G. duodenalis* was classified as assemblage AII at the *tpi* locus and assemblage AI at the *bg* and *gdh* loci (Figure 2A–C), suggesting that the isolate might exhibit zoonotic properties.

### 4.2. Whole-Genome Resequencing Analysis

Bioinformatics analysis of the whole-genome resequencing data from the isolate yielded a raw dataset and was compared to the genomes of other isolates. For the isolated *G. duodenalis* (as GF1), whole-genome resequencing generated a total of 21,055,176 clean reads, with an average read depth of 138.56× for each accession. The genome size was 12.1 Mbp, and the average guanine–cytosine (GC) content was 56.28%.

**Figure 3:**
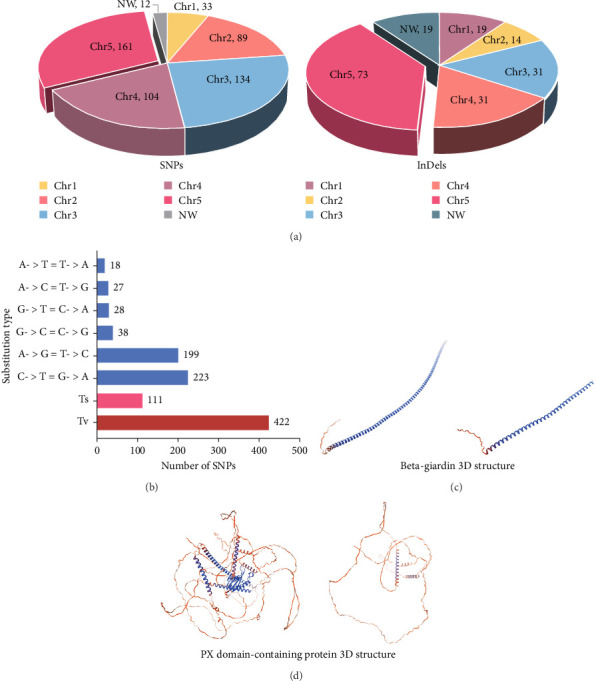
Genomic data of the isolates. (A) The numbers of SNPs (top panel) and InDels (bottom panel) on each chromosome. (B) Numbers of transition (Ts) and transversion (Tv) SNPs identified. (C, D) The tertiary structures of beta-giardin and PX domain-containing proteins were predicted using SWISS-MODEL software. (C) The structural predicted of *G. duodenalis* beta-giardin protein. (Left panel: *Giardia* assenblage A isolate WB. Right panel: *Giardia* assenblage A isolate ferret.) (D) The structural predicted of *G. duodenalis* PX domain-containing protein. (Left panel: *Giardia* assenblage A isolate WB. Right panel: *Giardia* assenblage A isolate ferret).

Compared with *Giardia* isolates from other hosts, the genome of GF1 was relatively large and had a high GC content, indicating its unique genetic composition (Table 1). Furthermore, we identified 720 variants shared by the isolate, including 533 SNPs and 187 insertions/deletions (InDels), in the whole-genome resequencing data compared to the genome of *G. duodenalis* WBC6 (Supporting Information Figure S1). Most variants were mapped to chromosome 5 (Figure 3A). All SNPs were divided into 6 classes, of which 111 (20.8%) were of transition (Ts) type, and 422 (79.2%) were of transversion (Tv) type, resulting in a Ts/Tv ratio of 0.26 (Figure 2B). In GF1, high-impact variants consisting of 19 variants were prioritized in this study (Supporting Information Table S2). Of these, 14 InDels caused frameshift variants, and 5 SNPs caused stop gains. Specifically, in the genes in which frameshift variants occurred, one gene (GL50803_00137688) exhibited insertions or deletions at four different positions (c.2559_2560insAC, c.2561_2562delAG, c.2891_2894delACGC, and c.2899_2900insGGGC), which is predicted to result in a complete alteration in the corresponding protein (Supporting Information Table S2).

Among the genes with stop-gain variants, we found that the *Giardia*-specific cytoskeletal protein-encoding gene beta-giardin (GL50803_004812) [21] harbored a premature stop codon at position 397 (c.397C >T) (Supporting Information Figure S2), leading to the truncation of the protein to ~49% of the reference sequence length (Figure 3C). PX domain-containing proteins play roles in various cellular processes [22], such as vesicle docking, cell survival, and signal transduction. The gene encoding a PX domain-containing protein (GL50803_0042357) on chromosome 3 exhibited a premature stop codon at position 1237 (c.1237G >T) (Supporting Information Figure S3), predicted to truncate the protein to about 40% of the reference sequence length (Figure 3D).

### 4.3. Variant Gene Annotation

**Figure 4:**
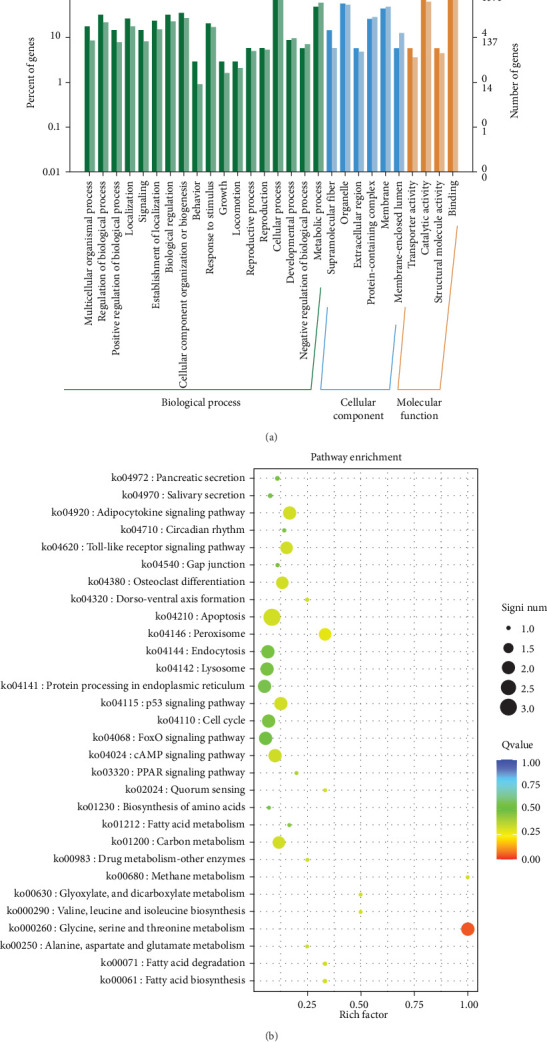
(A) GO and (B) KEGG pathway annotation analysis of variant genes.

Variant gene annotation analyses were conducted using multiple databases, including Gene Ontology (GO) and Kyoto Encyclopedia of Genes And Genomes (KEGG), to verify the functional characteristics of the genes. As shown in Figure 4, the variant genes of the isolated *G. duodenalis* were annotated into three main categories using the GO platform: biological process, cellular component, and molecular function. Biological processes were further classified into 18 subcategories, with the highest genes enrichment observed in cellular processes (GO:0009987) and metabolic processes (GO:0008152). Within the cellular components, organelles (GO:0043226) and membranes (GO:0016020) were the most enriched in 6 subcategories. In the molecular function category, only four subcategories were annotated, with significant enrichment in binding (GO:0005488) and catalytic activity (GO:0003824) (Figure 4A). In the KEGG classification system, the variant genes were mapped to 33 metabolic pathways and classified into 5 functional categories. Among all categories, metabolic pathways were the most frequently annotated, with significant enrichment observed in glycine, serine, and threonine metabolism pathways (ko00260) (Figure 4B).

### 4.4. Pathogenicity of the Isolated *G. duodenalis*

The pathogenicity of the isolated *G. duodenalis* was evaluated in *vivo* using a gerbil model. No trophozoites or cysts were observed in any of the animals in 5 × 10^4^ and 1 × 10^5^ groups) at 7 dpi and in 5 × 10^4^, 1 × 10^5^, and 5 × 10^5^ groups at 14 dpi. With increasing infective dose (5 × 10^5^, 1 × 10^6^, 5 × 10^6^, 1 × 10^7^ groups at 7 dpi, 1 × 10^6^, 5 × 10^6^, 1 × 10^7^ groups at 14 dpi), trophozoites or cysts were observed and the infection rate increased (Table 2).

**Figure 5:**
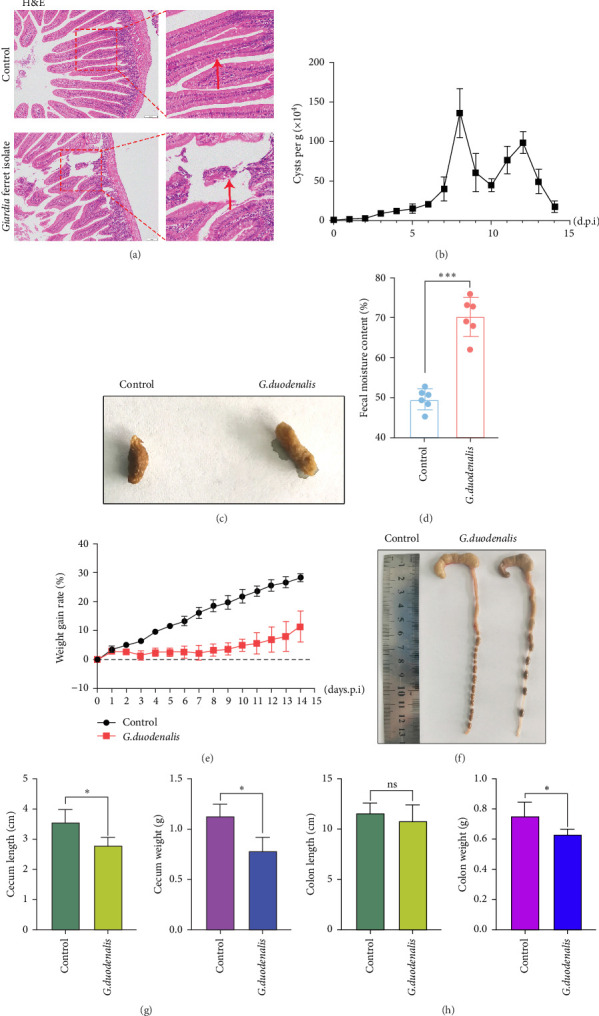
Pathogenicity of the isolate. (A) Pathological changes of duodenum according to H&E staining. (B) Shedding of *G. duodenalis* cysts during the experiment. (C) Comparative fecal appearance between control and *G. duodenalis*-infected groups. (D) Fecal moisture content analysis. (E) Weight gain rate over the course of infection. (F) Morphological observation of large intestine tissue of gerbil. (G) Quantification of cecum and colon weights. (H) Measurements of cecum and colon length. *⁣*^*∗*^*p*  < 0.05, *⁣*^*∗∗∗*^*p*  < 0.001, ns: not significant.

To examine the changes of cyst shedding, gerbils were gavaged at a dose of 1 × 10^7^ to monitor cyst shedding in the feces from 0 to 14 dpi, and the duodenum was analyzed histologically using H&E staining. Significant pathological injury was demonstrated following infection, including atrophied and broken duodenal villi and shallow crypts (Figure 5A). The results showed that the cysts were detected at day 3 dpi, but were very few. Thereafter, shedding gradually increased, with peak of cyst shedding occurring on day 8 post-infection (Figure 5B). In the infected group, no significant watery diarrhea or mortality was observed in gerbils. Compared with the control group, the feces of gerbils in the infected group were yellowish in color and relatively moist from 7 to 12 dpi. At 8 dpi, the feces were significantly softer, with a moisture content 1.4-fold higher than that of the control group (Figure 5C, D). Additionally, the results showed that the gerbils in the infected group gained weight slowly, with a significantly lower rate of weight gain compared to the control group (Figure 5E). In addition to the duodenal lesions described above, we conducted a systematic histopathological assessment of the jejunum, ileum, cecum, and colon. We found villous atrophy and lymphocytic infiltration in the jejunum and ileum (Supporting Information Figure S4A and B), but not as severe as in the duodenum. However, only a few trophozoites could be observed in the jejunum. while the cecum and colon showed mucosal hyperplasia and mild inflammation (Supporting Information Figure S4C and D). In addition, we conducted statistical analyses on the weight and length of the cecum and colon (Figure 5F). The results showed that the weight and length of the cecum in the infected group were significantly reduced (Figure 5G). The weight of the colon decreased, while there was no significant difference in length (Figure 5H).

### 4.5. Prevalence of *G. duodenalis* in Ferrets

To further determine the prevalence of *G. duodenalis* in ferrets, we detected and analyzed a total of 111 ferret fecal samples by amplifying three loci (*tpi*, *gdh*, *bg*). Sixteen samples tested positive for *G. duodenalis*, and a positive rate of 14.41% (95% CI: 7.8%−21.1%). Four of these samples were positive for pet ferrets in Jilin province, with a positive rate of 36.3% (95% CI: 2.5%−70.3%), and 12 samples were positive for a farm in Shandong province, with a positivity rate of 12% (95% CI: 5.5%−18.5%) (Table 3).

Gene typing of the 16 *Giardia*-positive samples was conducted according to *tpi*, *bg*, and *gdh* locus sequences, and results showed 12 *tpi*, 9 *bg*, and 1 *gdh* locus sequences. Only one sample was successfully sequenced across all three loci. Sequence analysis revealed that all 16 positive samples were classified as assemblage A of *G. duodenalis* (Table 3).

### 4.6. Genotypic Features of *G. duodenalis* in Ferrets

To gain further insight into the genotypic features, we performed sequence alignment according to *tpi*, *bg*, and *gdh* locus sequences. For the 12 *tpi* locus, we detected 9 polymorphic sites within the 468 bp region of the reference sequence (L02120) according to Yaoyu Feng [12] and Lihua Xiao (Table 4). JL-7, SD-30, SD-36, and SD-52 exhibited an identical genetic profile to the reference sequence (L02120). Additionally, SD-9, SD-10 and SD-11 exhibited identical genetic profiles but differed from the L02120 sequence by only 1 nucleotide (A136T). SD-2 and SD-12 displayed substitutions at positions: T19C, G33A, T94C and A202G. The remaining samples exhibited 3–5 nucleotide differences (Table 4). 6 of 12 *tpi* locus exhibited 100% similarity to isolates from various mammals, including humans. Among these, JL-5, JL-7, and SD-12 were identical to isolates obtained from humans (EU041753, PP584163, and EU041756). Notably, JL-7 also showed 100% similarity to an isolate collected from a ferret in Japan (AB509384). SD-30, SD-36, and SD-52 were identical to those obtained from a diverse array of mammals including donkeys (MN704937.1), horses (MF169203), sheep (MK442911, MK473861, JQ688289), musk deer (MF497412), dog (KP780971), and calf (AB781151). Interestingly, SD-44 and JL-11 exhibited unique sequences that had not been previously reported.

For the 9 *bg* locus, 15 polymorphic sites were identified compared to the reference sequence (X85958). Additionally, 2–6 nucleotide differences were detected in each sample. Most samples (*n* = 5) exhibited substitutions at positions 973 (X85958, C973T) and 1222 (X85958, C1222T). However, 5 samples (JL-5, JL-7, SD-1, SD-22, and SD-52) and 4 samples (JL-5, JL-7, SD-1, and SD-22) showed deletions at positions 953 and 962, respectively, which have not been reported previously (Table 5). SD-1, SD-52, JL-5, JL-7, JL-9, and JL-11 exhibited novel sequences, while only one sample (SD-2) aligned with the previously reported reference sequence at 100% identity. Interestingly, this isolate was identical to those obtained from humans in Sweden (GQ329671) and from ferrets in Japan (AB508814).

For the *gdh* locus, a positive sample (JL-11) was classified as belonging to assemblage A. Comparison with the reference sequence revealed a nucleotide substitution at position 797 (EF507642, C797T) and the insertion of two nucleotides between 801 and 802.

### 4.7. Phylogenetic Analysis

**Figure 6:**
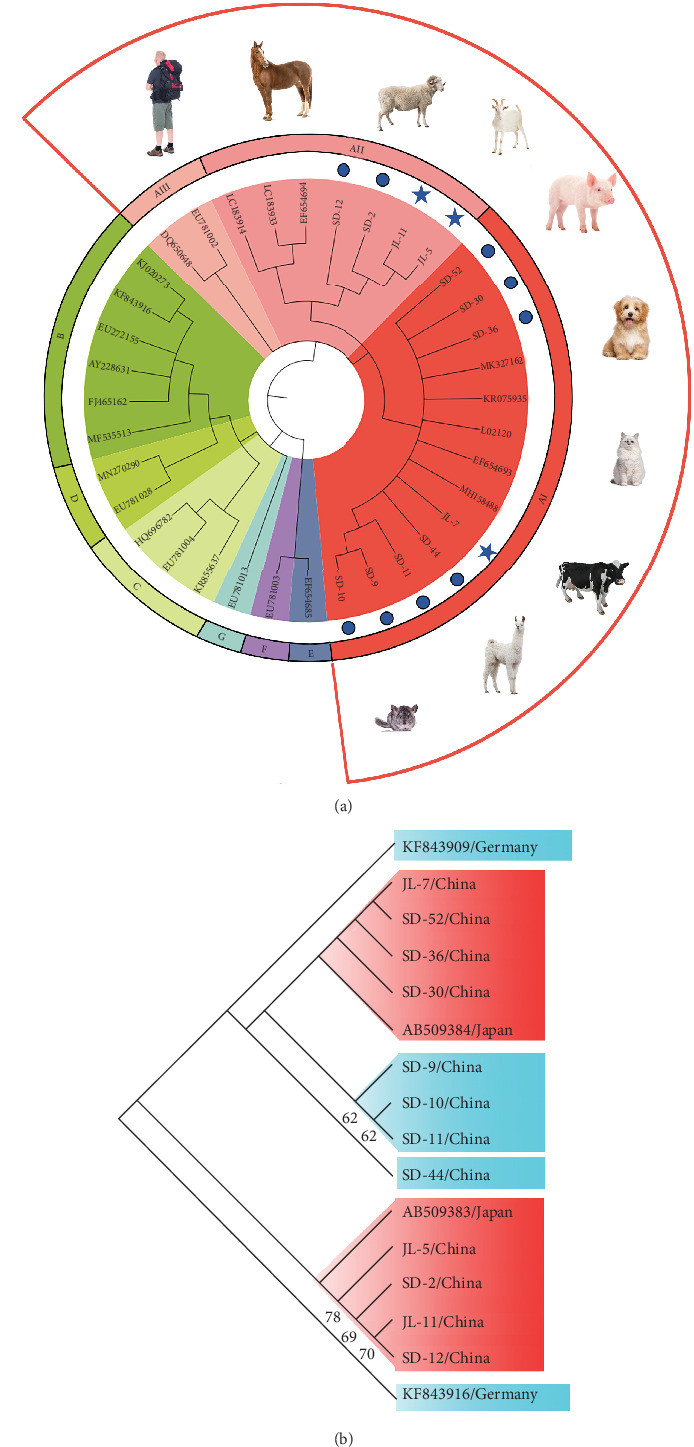
The phylogenetic tree based on analysis of *tpi*. (A) Phylogenetic analysis of *G. duodenalis* sequences detected in this study and previously published from GenBank. Filled circles indicate assemblages obtained from farmed ferrets in Shandong province, and filled pentagrams indicate assemblages obtained from pet ferrets in Jilin province in this study. (B) Phylogenetic analysis of *G. duodenalis* sequences detected in this study and previously published from ferrets.

In order to situate the obtained gene sequences within a broader phylogenetic context, we conducted phylogenetic analyses. As shown in Figure 6A, for the *tpi* locus, eight positive samples (JL-7, SD 9–11, SD-30, SD-36, SD-44, and SD-52) of *G. duodenalis* were clustered with assemblage AI, whereas four samples (JL-5, JL-11, SD-2, and SD-12) were clustered with assemblage AII. Four isolates (JL-5, JL-11, SD-2, and SD-12) clustered into a single branch with AB509383 stain obtained from a ferret in Japan, indicating a close phylogenetic relationship (Figure 6B). Similarly, the other four isolates (JL-7, SD-30, SD-36, and SD-52) were closely related phylogenetically to isolate AB509384 strain. However, the isolates in this study exhibited a relatively distant phylogenetic relationship with isolates (KF843909 and KF843916) obtained from ferrets in Germany (Figure 6B).

In addition, for the *bg* locus, *Giardia*-positive samples clustered into three subtypes, with assemblage AI clustering into the most numerous (Supporting Information Figure S5). Furthermore, two isolates (SD-2 and SD-6) were closely related phylogenetically to isolate AB159797 and AB508814 strains obtained from ferrets in Japan (Supporting Information Figure S6A). However, isolate JL-11 showed a relatively distant phylogenetic relationship with three isolates (AB159795, AB469364, and AB508813) from ferrets in Japan at the *gdh* locus (Supporting Information Figure S6B).

## 5. Discussion

In this study, we isolated a novel *G. duodenalis* from a diarrheal pet ferret in China and systematically characterized its morphological, evolutionary, and pathogenic properties. This is the first comprehensive investigation of *G. duodenalis* in ferrets. Furthermore, the epidemiological survey conducted on both pet and farmed ferrets provided valuable data on the prevalence and distribution of *Giardia* infections in these populations.

*G. duodenalis* comprises eight distinct assemblages (A–H) with varying host specificities [23]. The study of *G. duodenalis* species is becoming more systematic, but morphological studies of isolates are easily neglected. Currently, isolates of *G. duodenalis* from human and livestock have been examined [24], but little is known about isolates from ferrets. In this study, we have obtained cysts from a ferret feces and successfully cultured *G. duodenalis* isolates *in vitro*. The morphology of the isolated cysts and its trophozoites was consistent with the characteristics of *G. duodenalis* [25]. Subsequently, this isolate was clustered into assemblage A at the *tpi*, *bg*, and *gdh* loci by molecular typing, considered as a potential zoonotic risk. Differently, the isolate was classified as assemblage AII at the *tpi* locus, whereas at the *gdh* and *bg* loci, it was classified as assemblage AI. This appears to be the first time that assemblage AII has been identified in ferrets, as only assemblage AI and BIV have been detected in a few previous isolates [26, 27]. In addition, our results first provided direct evidence of mixed-assemblage infection in ferrets, which are common in both humans and animals [12]. Previous studies have shown that mixed assemblages occur in animal samples from cats, cattle, pigs, dogs, and beavers [9, 28–30]. In beavers, mixed assemblages A/B were present in five samples, and mixed assemblages AI/AII were present in one sample [28]. In some dogs, three mixed-assemblages could be observed, such as assemblages A/B/C or B/C/D [30]. It has been reported that assemblages-specific detection provided more accurate assessment of the occurrence of mixed infections. Thus, our data will improve our understanding of the identification and differentiation of *G. duodenalis* in ferrets, and indicating that the diversity of *G. duodenalis* in ferrets is complex, and more work will be needed. Besides, given the possibility of genetic exchange between isolates of assemblage A, and even between assemblages A and B, further studies are needed to distinguish between recombination or mixed infection.

Recent studies have shown that mixed infections of different assemblages could enhance intestinal cells damage *in vitro*, underestimating the importance of mixing [31]. In this study, our results revealed key insights into the analysis of pathogenicity, dose–response relationships, and persistence of infection in this mixed isolate, providing valuable data to support the pathogenicity of mixed isolates of *G. duodenalis*.

Currently, comprehensive genomic data of assemblage AI, AII, B, and E isolates from humans, pigs, beavers, and other sources have been obtained through whole-genome sequencing, providing strong evidence for distinguishing different *Giardia* species [32–34]. Hence, in our study, the whole-genome resequencing and annotation study on this mixed isolate obtained from ferret confirmed that this *G. duodenalis* is a novel isolate, which is larger than that of isolates from human, cat, pig and beaver, but smaller than that of dog isolates. This could be due to the mixture of assemblage AI and AII in the isolate, but further systematic analyses are needed in the future study. Furthermore, this mixed isolate was characterized by high GC content, Tv values, and low Ts/Tv, which were different from other isolates. In addition, 19 high-impact variants were identified, including 14 frameshift variants and 5 stop gains. These variants may affect the biological functions and phenotypic traits of *G. duodenalis*. Beta-giardin is a component of *G. duodenalis* ventral disk, which plays a key role in trophozoite attachment to host epithelial cells [35]. Previous studies have demonstrated that beta-giardin mRNA levels are strongly correlated with the viability of the cysts in the environment [36]. Moreover, studies have shown that mutations in the beta-giardin gene play an important role in albendazole resistance, indirectly indicating that beta-giardin mutations are closely related to the pathogenicity of *G. duodenalis* [37]. In the current study, the beta-giardin of the new isolate contained a stop codon at position 397, resulting in a significantly different amino acid sequence and tertiary structure. GO functional annotation enrichment analysis indicated that the beta-giardin was involved in structural constituent of cytoskeleton. Therefore, further experiments are needed to verify its difference from other *G. duodenalis* strains and its effect on pathogenicity. Another variant gene, argonaute (GL50803_002902), plays a key role in *G. duodenalis* proliferation, as its knockdown has been shown to inhibit trophozoites growth [38]. GO enrichment analysis in this study revealed associations of argonaute with RNA-mediated gene silencing, regulation of nitrogen metabolism, and cellular metabolic processes, providing insights into its mechanistic role in trophozoites proliferation. Additionally, several previously uncharacterized high-impact variant genes were identified, and their potential relevance to pathogenicity, host adaptation, or zoonotic potential will be explored in future studies. This study provides new insight into the genetic differences and molecular characteristics of the isolate, and expands the availability of *G. duodenalis* genomes from infected hosts.

Currently, epidemiological data on *G. duodenalis* in ferrets are very limited, with reports from only a few countries (Supporting Information Table S3). In Japan, *G. duodenalis* was detected in three domestic ferrets, all identified as assemblage A [26, 39]. A study carried out in the UK showed a significant increase in infection rate, from 2.9% to 13.3%, though no genetic typing was conducted [18]. In another European study, assemblages A and B were found in ferrets [27], but no reports existed in China before the present study. In this study, the overall prevalence of *G. duodenalis* in ferrets was 14.41%. The infection rate was higher rate in household pets (36.3%) compared to farmed ferrets (12%). These *G. duodenalis* samples were identified as assemblage A, which indicated the zoonotic potential. Our results were inconsistent with previous reports from Japan and several European countries and showed higher infection rate than in the UK [18]. Different detection methods, sample sizes and inclusion criteria may explain the differences in results. Despite the high infection rate in pet ferrets due to low sample size and other reasons, the transmission of *G. duodenalis* should not be ignored [40]. Humans and dogs in a remote Indian community were found to have similar *G. duodenalis* assemblages [41]. Cats and dogs may also transmit giardiasis [42]. These findings suggest the potential for zoonotic transmission between humans and animals. Therefore, it is crucial to regularly monitor pets and domestic animals to prevent potential infections that could be transmitted from these animals to humans. Our study provided a better understanding of *G. duodenalis* epidemiology in ferrets from Shandong and Jilin provinces. There is no denying that there are some limitations and deficiencies in this study. The limited sample size and geographic scope might affect the generalizability of the results. Therefore, for future investigations, we will aim to collect samples from a broader geographic range in China, particularly including southern, western, and central regions. This will help us gain a more comprehensive understanding of *G. duodenalis* epidemiology in ferrets in China and potentially reveal regional variations in prevalence, assemblage distribution, and zoonotic potential.

*G. duodenalis* assemblage A has been found to infect humans [43, 44] and livestock (e.g., cattle, sheep, goats, and pigs) [11, 23, 45] as well as companion animals (e.g., dogs and cats) [46, 47]. In this study, assemblage A was the only genotype detected in ferrets, and all 12 *tpi*, 9 *bg*, and 1 *gdh* gene locus sequences belonged to assemblage A. These results are consistent with previous reports from Japan [39], but not with reports from Germany [27], which found assemblages A and B at three loci (*ssu*, *tpi*, *bg*) in ferret isolates. Our findings suggested that assemblage A may be a widely distributed dominant genotype in ferrets in China. Notably, eight novel genotypic sequences were identified in ferrets, which significantly improves our understanding of the genetic diversity of *G. duodenalis*. Therefore, the positive samples obtained in this study might not be ferret-specific, but might have a greater potential for zoonotic transmission. Additionally, phylogenetic analyses revealed that the isolates in this study from China were phylogenetically more closely related to isolates from Japan, but more distantly related to isolates from Germany. This may reflect regional zoonotic transmission dynamics that differ from those in Europe. In East Asia, companion animals (e.g., cats and dogs) and wildlife (e.g., wild sika deer) are increasingly implicated in the transmission of *G. duodenalis*, especially the assemblages A and B [12, 48]. The phylogenetic relationship with the Japanese isolates could be further influenced by anthropogenic factors, such as the international pet trade that facilitates cross-border movement of infected animals. The significant pet trade between our study regions and Japan may amplify parasite transmission. Future studies need to combine genomic epidemiology with animal movement data to confirm these hypotheses. Nevertheless, these results contribute significantly to the understanding of the genetic diversity and transmission dynamics of *G. duodenalis* in ferrets and imply the zoonotic risk.

## 6. Conclusion

In conclusion, a novel *G. duodenalis* from a ferret was isolated for the first time with assemblage of mixed AI/AII, and the genome size, GC content, and Ts/Tv ratio of this mixed isolate were significantly different from those of other isolates. The mixed isolate was pathogenic to gerbils *in vivo*, and the cyst of this isolate can be excreted from infected gerbils, providing further understanding of the pathogenicity and transmission dynamics of this *G. duodenalis*. Additionally, the overall prevalence of *G. duodenalis* in ferrets from Shandong and Jilin provinces was 14.41%, with assemblage A as the dominant genotype. This study supplements the occurrence and epidemiological data of *G. duodenalis* in ferrets in China and may provide broader understanding of zoonotic transmission of *G. duodenalis* in ferrets and contribute to the efforts to control parasitic and zoonotic diseases globally. Nevertheless, broader geographic sampling is essential to fully characterize the epidemiological landscape of *G. duodenalis* in ferret populations nationwide.

## Figures and Tables

**Table 1:** Comparative analysis of genome characteristics in *G. duodenalis* isolates from different hosts.

	GF1	DH	GS	CIA	DID	P15	Beaver
Host	Ferret	Human	Human	Cat	Dog	Pig	Beaver
Assemblage	A	A	B	A	D	E	—
Size (Mbp)	12.1	10.7	12	10.7	13.1	11.5	11.5
Depth	138.56X	124.0X	53.0X	55.0X	254.0x	47.0X	245.0X
GC Bases Ratio (%)	56.3	49	48	49	41	47	49.5
No. of Chromosomes	5	5	5	5	5	5	5

*Note:* GF1: *Giardia duodenalis* ferret isolate.

**Table 2:** Infection of gerbils by *Giardia* ferret isolates.

	Doses of infectious (× 10^5^)					
	0.5	1	5	10	50	100
7 days p.i.	0 (0/5)	0 (0/5)	20% (1/5)	40% (2/5)	80% (4/5)	100% (5/5)
14 days p.i	0 (0/5)	0 (0/5)	0 (0/5)	20% (1/5)	60% (3/5)	100% (5/5)

**Table 3:** Prevalence and genotype distribution of *G. duodenalis* in ferrets from different regions of China.

Province	Fecal samples	No. of specimens	No. positive (%)	95% CI (%)	Assemblage (no. of samples)						
					*tpi*	*bg*	*gdh*	*tpi/bg*	*tpi/gdh*	*gdh/bg*	*tpi/gdh/bg*
Jilin	Pet shop	11	4 (36.3)	2.5–70.3	A (3)	A (4)	A (1)	A/A (3)	A/A (1)	A/A (1)	A/A/A (1)
Shandong	Breeding farm	100	12 (12.0)	5.5–18.5	A (9)	A (5)	N	A/A (2)	N	N	N

*Note:* N, not amplified.

**Table 4:** Variations in the *tpi* nucleotide sequences of *G. duodenalis* isolated from ferrets in this study.

Isolates ID	Assemblage	Nucleotide at position									No. of differences with reference					Reference
		19	23	30	33	94	136	202	241	451						
	—	T	T	A	G	T	A	A	G	G	—					L02120 (position 563 to 1030)
JL-5	A	·	·	·	A	C	·	G	·	·	3					—
JL-11	A	C	·	·	A	C	·	G	·	-	5					—
SD-2	A	C	·	·	A	C	·	G	·	·	4					—
SD-9	A	·	·	·	·	·	T	·	·	·	1					—
SD-10	A	·	·	·	·	·	T	·	·	·	1					—
SD-11	A	·	·	·	·	·	T	·	·	·	1					—
SD-12	A	C	·	·	A	C	·	G	·	·	4					—
SD-44	A	·	A	-	·	·	·	·	A	·	3					*—*

*Note:* Dots (**·**) denote nucleotides identical to those of the reference sequence. Dashes (-) denote missing nucleotides compared to the reference sequences.

**Table 5:** Variations in the *bg* nucleotide sequences of *G. duodenalis* isolated from ferrets in this study.

Isolates ID	Assemblage	Nucleotide at position															No.of differences with reference	Reference
		915	940	943	944	953	962	963	973	1038	1169	1222	1284	1307	1320	1413		
	A	A	C	C	A	A	A	A	C	C	C	C	T	G	T	-	—	X85958
JL-5	A	·	A	·	-	-	-	·	T	·	·	T	·	·	·	·	6	—
JL-7	A	·	·	·	·	-	-	T	·	·	·	·	·	·	·	·	3	—
JL-9	A	G	·	·	·	·	·	·	·	·	·	·	C	-	-	·	4	—
JL-11	A	·	·	·	·	·	·	·	T	·	·	T	·	·	·	T	3	—
SD-1	A	·	·	·	·	-	-	·	·	A	·	·	·	·	·	·	3	—
SD-2	A	·	·	·	·	·	·	·	T	·	·	T	·	·	·	·	2	—
SD-6	A	·	T	·	·	·	·	·	T	·	·	T	·	·	·	·	3	—
SD-22	A	·	·	·	·	-	-	·	T	·	·	T	·	·	·	·	4	—
SD-52	**A**	**·**	**·**	**G**	**·**	-	**·**	**·**	**·**	**·**	**T**	**·**	**·**	**·**	**·**	**·**	**3**	***—***

*Note:* Dots (.) denote nucleotides identical to those of the reference sequence. Dashes (-) denote missing nucleotides compared to the reference sequences.

## Data Availability

Both this manuscript and the supporting information contain publicly available data that back up the study's findings.

## Supporting Information

Supporting Information 1 Video S1: Microscopic observation of *G. duodenalis* trophozoites. This video shows *G. duodenalis* trophozoites in duodenal lavage fluid.

Supporting Information 2 Table S1: PCR primers used in this study.

Supporting Information 3 Table S2: High impact missense SNPs.

Supporting Information 4 Table S3: Prevalence and genotype distribution of *G. duodenalis* in ferrets based on published reports.

Supporting Information 5 Figure S1: Genomic circle diagram. From the outside circle to the inside circle are chromosomes, GC content, reads coverage depth, SNP,and InDel number distribution. Note: GF1: *G. duodenalis* ferret isolate.

Supporting Information 6 Figure S2: SNP in BG protein.

Supporting Information 7 Figure S3: SNP in PX domain-containing protein.

Supporting Information 8 Figure S4: Observation of pathological changes in intestinal tissue by HE staining. (A) Jejunum, (B) ileum, (C) cecum, and (D) colon.

Supporting Information 9 Figure S5: The phylogenetic tree based on analysis of bg.

Supporting Information 10 Figure S6: Phylogenetic analysis of *G. duodenalis* sequences detected in this study and previously published from ferrets. (A) The phylogenetic tree based on analysis of bg. (B) The phylogenetic tree based on analysis of gdh.
