# Supplementary material for: First Characterization and Zoonotic Potential Evaluation of Giardia duodenalis in Ferrets in China
Source: Transbound Emerg Dis. 2025 May 29;2025:3087035. doi: 10.1155/tbed/3087035 (PMC12140828; doi:10.1155/tbed/3087035)
Supplement: Supporting Information 2 — Table S1: PCR primers used in this study. [file 3087035.f2.docx]

| Gene | Primer ID | Primer sequences (5'-3') | Size | Reference |
| --- | --- | --- | --- | --- |
| *bg* | G7 | AAGCCCGACGACCTCACCCGCAGTGC | 753 bp | Sulaiman et al., 2003 |
|  | G759 | GAGGCCGCCCTGGATCTTCGAGAC GAC |  |  |
|  | BiarF | GAACGAACGAGATCGAGGTCCG | 511 bp |  |
|  | BiarR | CTCGACGAGCTTCGTGTT |  |  |
| *tpi* | AL3543 | AAATIATGCCTGCTCGTCG | 605bp |  |
|  | AL3546 | CAAACCTTITCCGCAAACC |  |  |
|  | AL3544 | CCCTTCATCGGIGGTAACTT | 532bp |  |
|  | AL3545 | GTGGCCACCACICCCGTGCC |  |  |
| *gdh* | GDHF1 | TTCCGTRTYCAGTACAACTC | 754 bp |  |
|  | GDHR1 | ACCTCGTTCTGRGTGGCGCA |  |  |
|  | GDHF2 | ATGACYGAGCTYCAGAGGCACGT | 530 bp |  |
|  | GDHR2 | GTGGCGCARGGCATGATGCA |  |  |

**Table S1: PCR primers used in this study.**

PCR was performed as follows: after an initial denaturation step of 15 min at 95 °C, a set of 35 cycles was run, each consisting of 30 sec at 95 °C, 30 sec of annealing (65 °C for the primary *bg* PCR, 55 °C for the nested *bg*, 52 °C for the primary *tpi*, 60 °C for the nested *tpi*, 55 °C for the primary *gdh* and 59 °C for the nested *gdh*) and 60 sec at 72 °C, followed by a final extension of 7 min at 72 °C.
