# Supplementary material for: First Characterization and Zoonotic Potential Evaluation of Giardia duodenalis in Ferrets in China
Source: Transbound Emerg Dis. 2025 May 29;2025:3087035. doi: 10.1155/tbed/3087035 (PMC12140828; doi:10.1155/tbed/3087035)
Supplement: Supporting Information 3 — Table S2: High impact missense SNPs. [file 3087035.f3.docx]

**Table S2:** **High impact missense SNPs**

| Protein Product | GENEID | CHROM | POS | REF | ALT | TYPE | IMPACT | EFFECT |
| --- | --- | --- | --- | --- | --- | --- | --- | --- |
| **Argonaute** | GL50803_002902 | Chr 1 | 372858 | G | A | SNP | HIGH | **stop_gained** |
| **5'-AMP-activated protein kinase, beta-1 subunit** | GL50803_00101919 | Chr 2 | 217228 | C | T | SNP | HIGH | **stop_gained** |
| Kinase, NEK | GL50803_0061439 | Chr 2 | 1529588 | TAG | T | INDEL | HIGH | frameshift_variant |
| Kinase, NEK | GL50803_0061439 | Chr 2 | 1529592 | A | ACT | INDEL | HIGH | frameshift_variant |
| Kinase, NEK | GL50803_0016988 | Chr 3 | 282589 | TC | T | INDEL | HIGH | frameshift_variant |
| Ciliary dynein heavy chain 11 | GL50803_0042285 | Chr 3 | 691896 | C | CG | INDEL | HIGH | frameshift_variant |
| **PX domain-containing protein** | GL50803_0042357 | Chr 3 | 1253588 | C | A | SNP | HIGH | **stop_gained** |
| Coiled-coil protein | GL50803_0015591 | Chr 3 | 1954988 | AGAAGCATCTTCTT | A | INDEL | HIGH | frameshift_variant |
| unspecified product | GL50803_0017580,XM_001707761.2 | Chr 4 | 810819 | G | A | SNP | HIGH,MODIFIER | stop_gained,intron_variant |
| **Beta-giardin** | GL50803_004812 | Chr 4 | 2037027 | C | T | SNP | HIGH | **stop_gained** |
| Trichohyalin | GL50803_0016840 | Chr 4 | 2365821 | CG | C | INDEL | HIGH | frameshift_variant |
| unspecified product | GL50803_0060194,XM_038045910.1 | Chr 5 | 1154419 | CT | C | INDEL | HIGH,MODIFIER | frameshift_variant,intron_variant |
| unspecified product | GL50803_0060194,XM_038045910.1 | Chr 5 | 1154421 | CAGCGTAGGAT | C | INDEL | HIGH,MODIFIER | frameshift_variant,intron_variant |
| TatD related Dnase | GL50803_0092892 | Chr 5 | 2571072 | A | AG | INDEL | HIGH | frameshift_variant |
| putative DNA polymerase | GL50803_00137688 | Chr 5 | 3017718 | G | GAC | INDEL | HIGH | frameshift_variant |
| putative DNA polymerase | GL50803_00137688 | Chr 5 | 3017719 | CAG | C | INDEL | HIGH | frameshift_variant |
| putative DNA polymerase | GL50803_00137688 | Chr 5 | 3018048 | CCACG | C | INDEL | HIGH | frameshift_variant |
| putative DNA polymerase | GL50803_00137688 | Chr 5 | 3018057 | C | CCGGG | INDEL | HIGH | frameshift_variant |
| Transmembrane domain-containing protein | GL50803_0060531 | Chr 5 | 3729106 | GT | G | INDEL | HIGH | frameshift_variant |
