# Supplementary material for: First Characterization and Zoonotic Potential Evaluation of Giardia duodenalis in Ferrets in China
Source: Transbound Emerg Dis. 2025 May 29;2025:3087035. doi: 10.1155/tbed/3087035 (PMC12140828; doi:10.1155/tbed/3087035)
Supplement: Supporting Information 4 — Table S3: Prevalence and genotype distribution of G. duodenalis in ferrets based on published reports. [file 3087035.f4.docx]

**Table S3: Prevalence and genotype distribution of *G. duodenalis* in ferrets based on published reports**

| **Year** | **Country** | **Prevalence(%)** | **Genotype(no. of samples)** | **Reference** |
| --- | --- | --- | --- | --- |
| 2005 | Japan | **—** | **A(1)** | Abe *et al.* 2005; |
| 2010 | Japan | **—** | **A(2)** | Abe *et al.* 2010 |
| 2011 | England | 2.9  13.3 | **—** | Pantchev *et al.* 2011 |
| 2014 | Italy | 0 | **—** | d'Ovidio *et al.* 2014 |
| 2014 | Germany | **—** | **A(2)/B(4)** | Pantchev *et al.* 2014 |
