# Supplementary figures and images for: First Characterization and Zoonotic Potential Evaluation of Giardia duodenalis in Ferrets in China
Source: Transbound Emerg Dis. 2025 May 29;2025:3087035. doi: 10.1155/tbed/3087035 (PMC12140828; doi:10.1155/tbed/3087035)

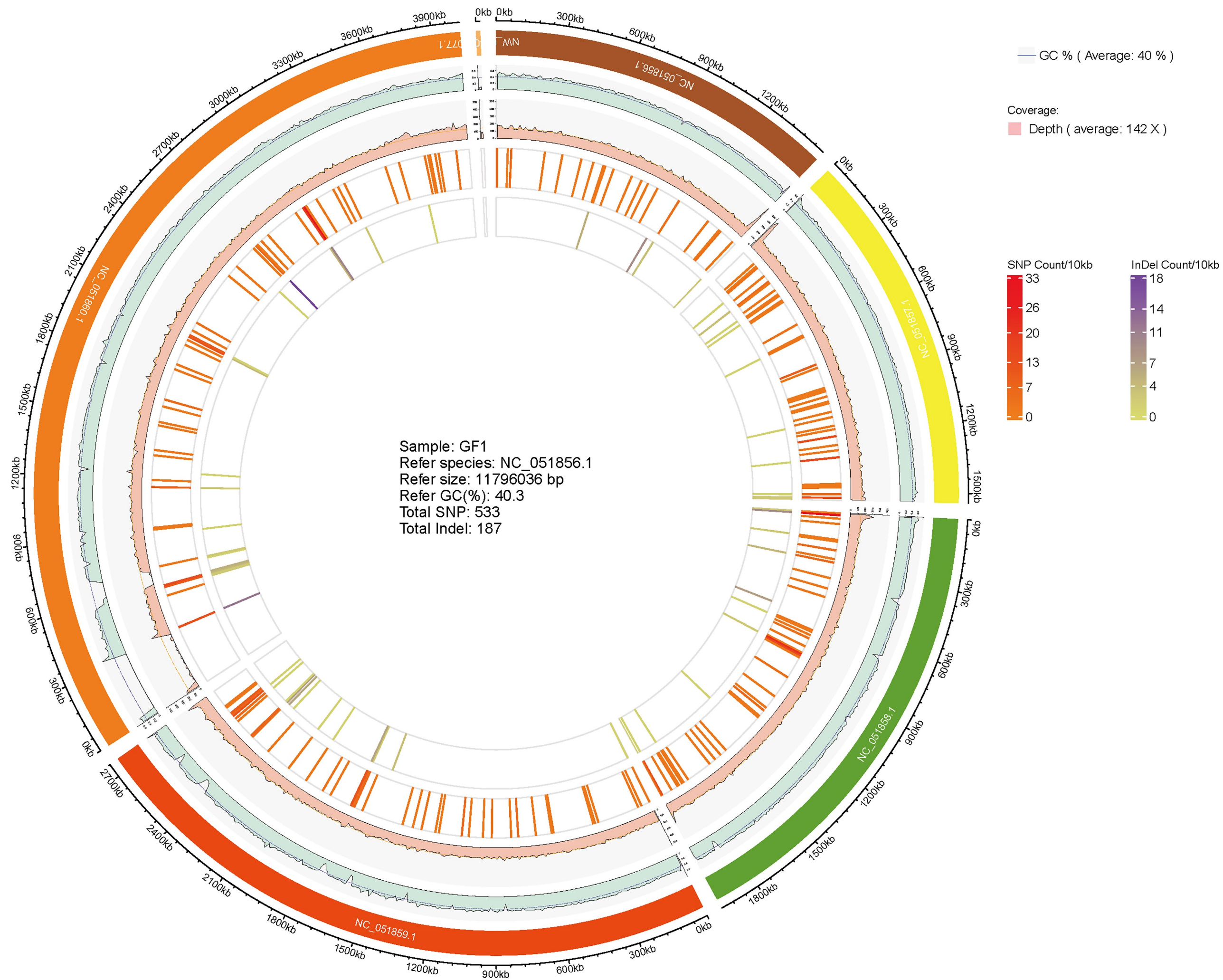

Supplement: Supporting Information 5 — Figure S1: Genomic circle diagram. From the outside circle to the inside circle are chromosomes, GC content, reads coverage depth, SNP,and InDel number distribution. Note: GF1: G. duodenalis ferret isolate. [file 3087035.f5.pdf]

Giardia Assemblage A isolate WB 2019

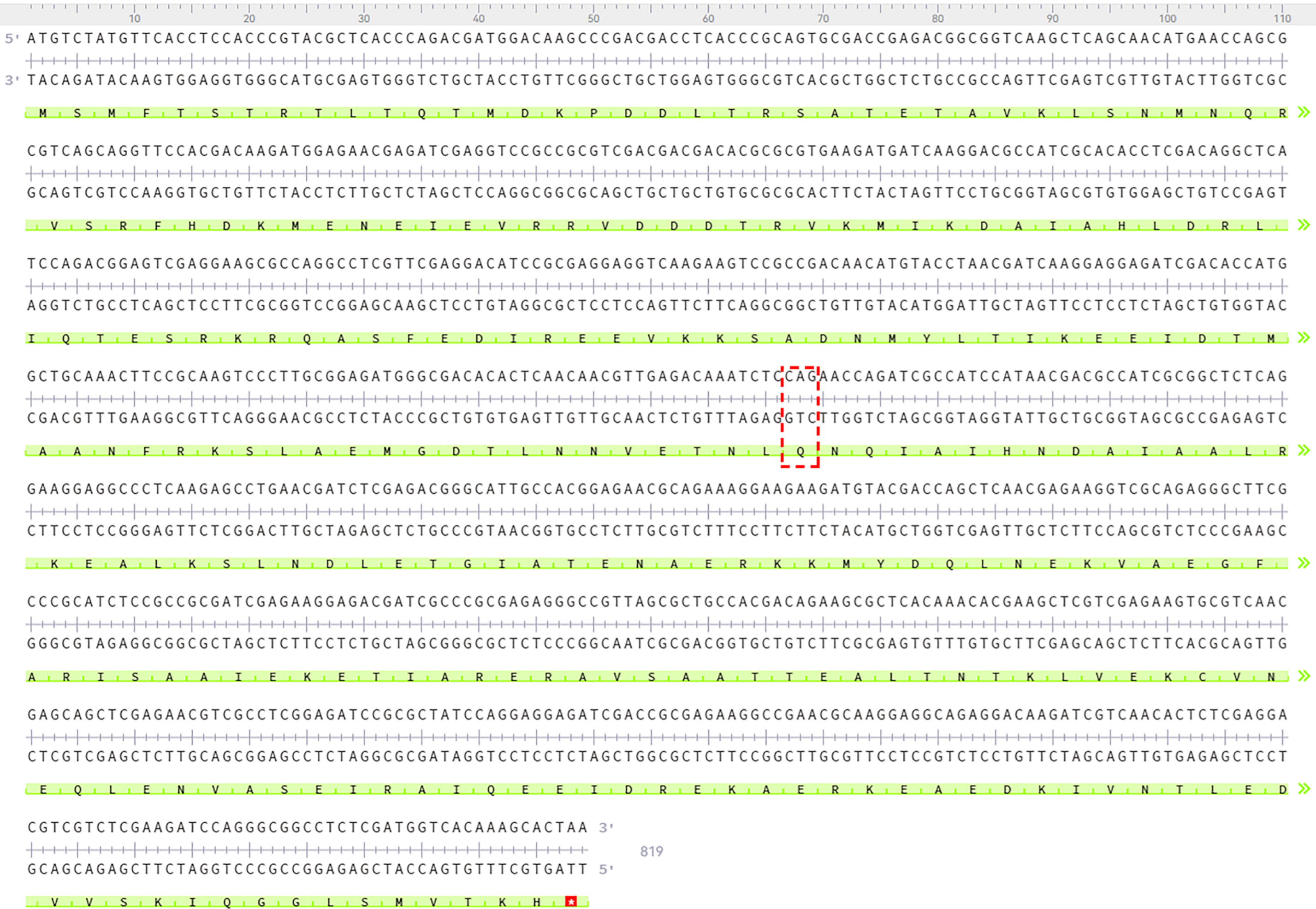

Giardia Assemblage A isolate Ferrest

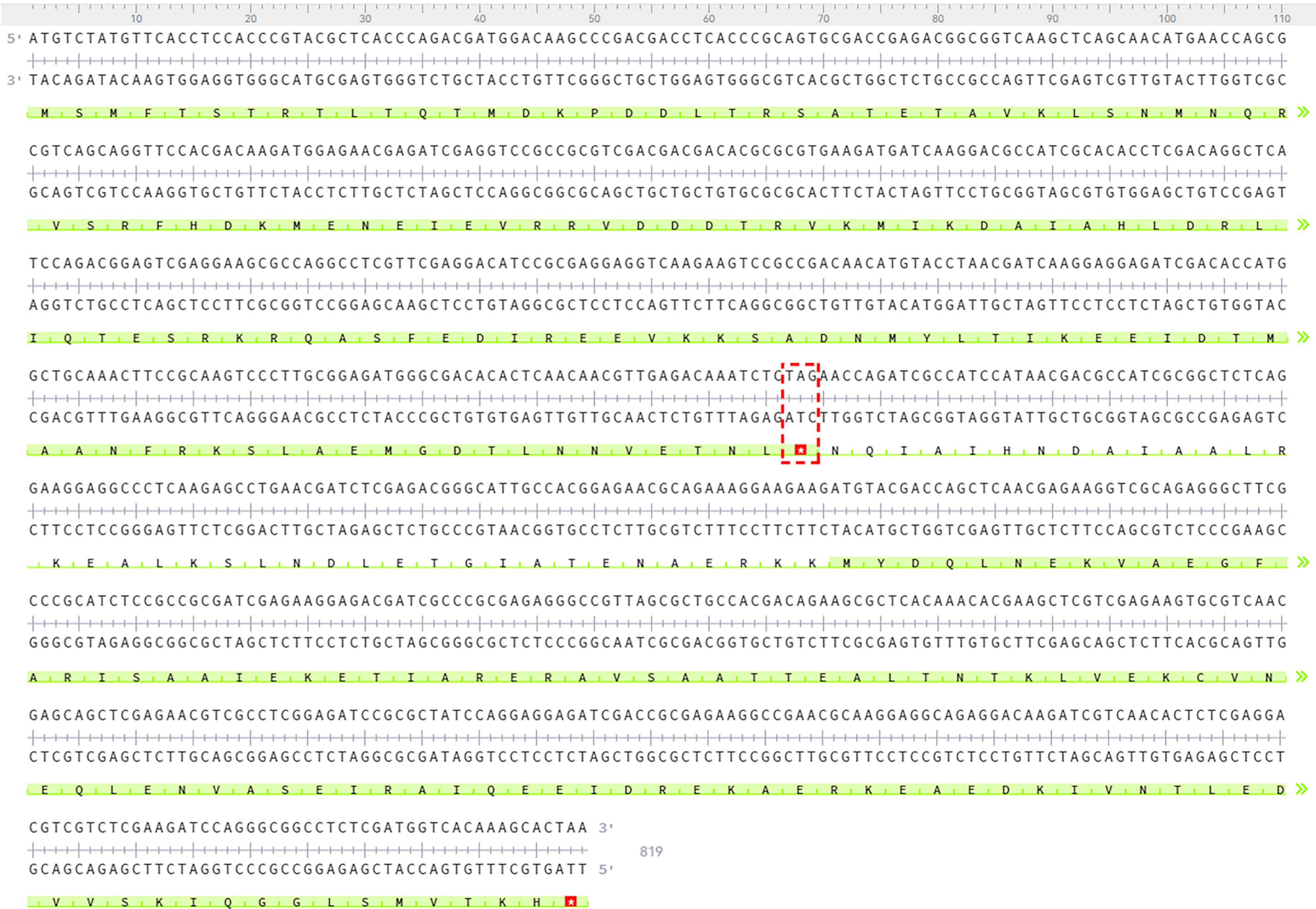

Supplement: Supporting Information 6 — Figure S2: SNP in BG protein. [file 3087035.f6.pdf]

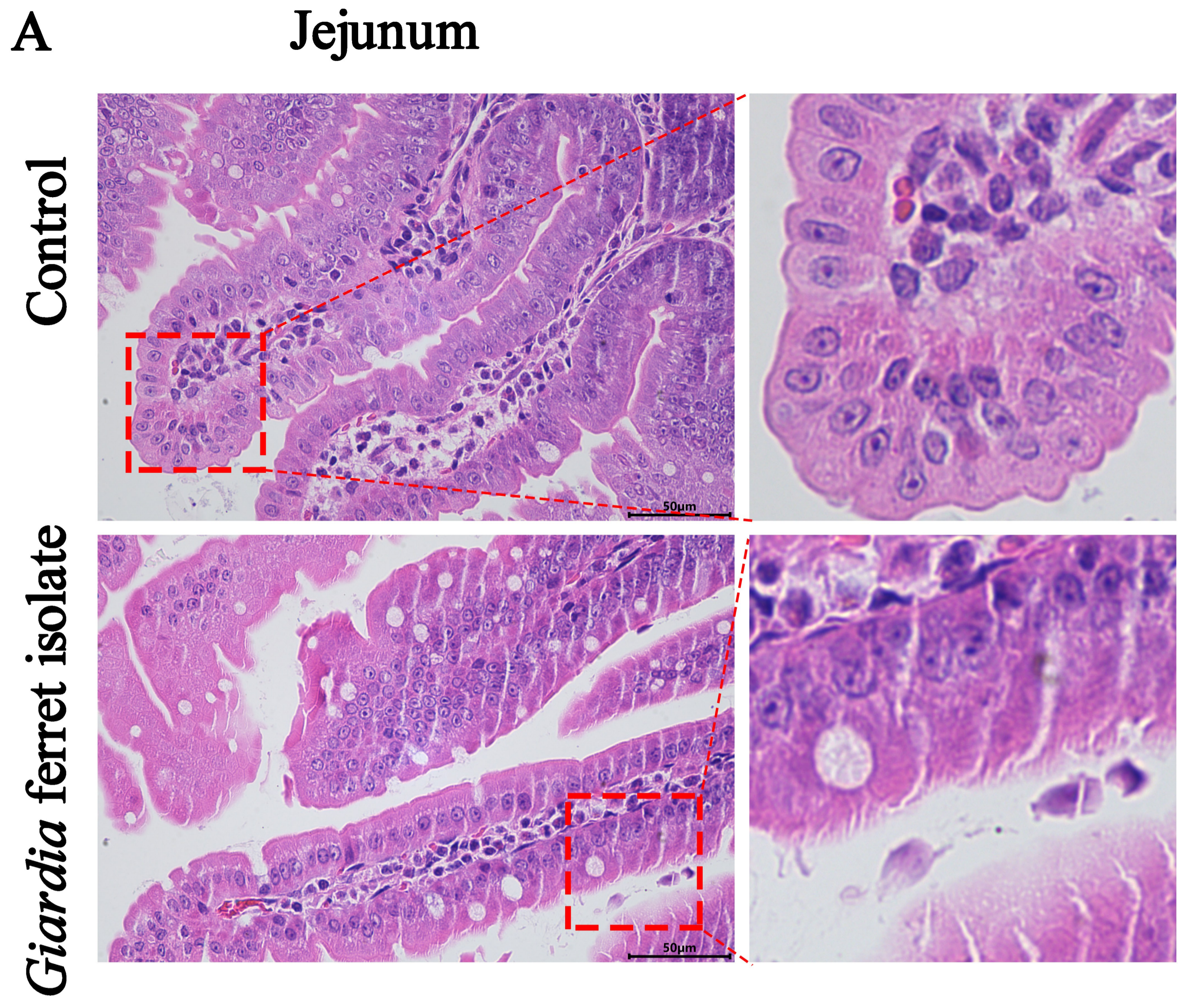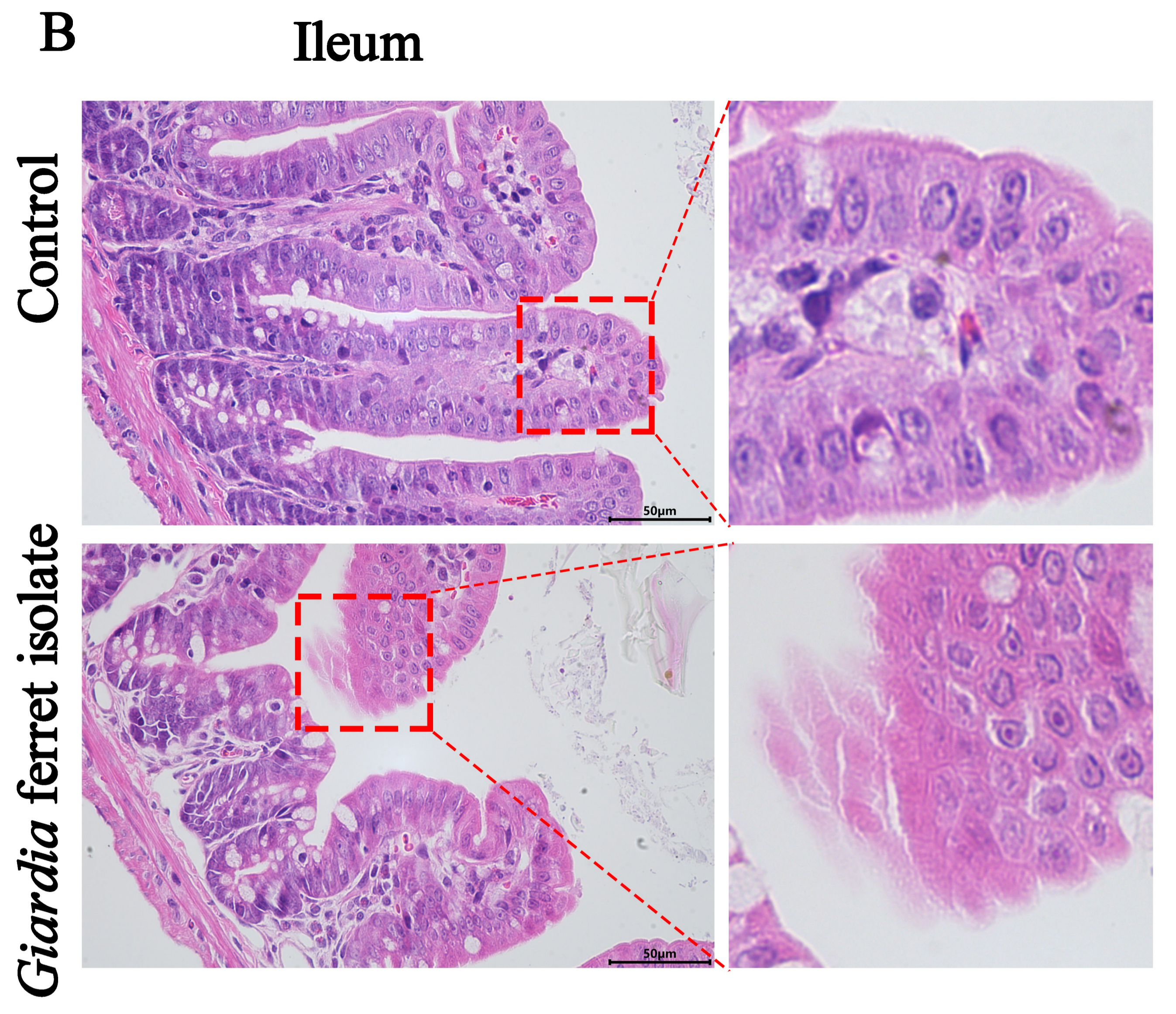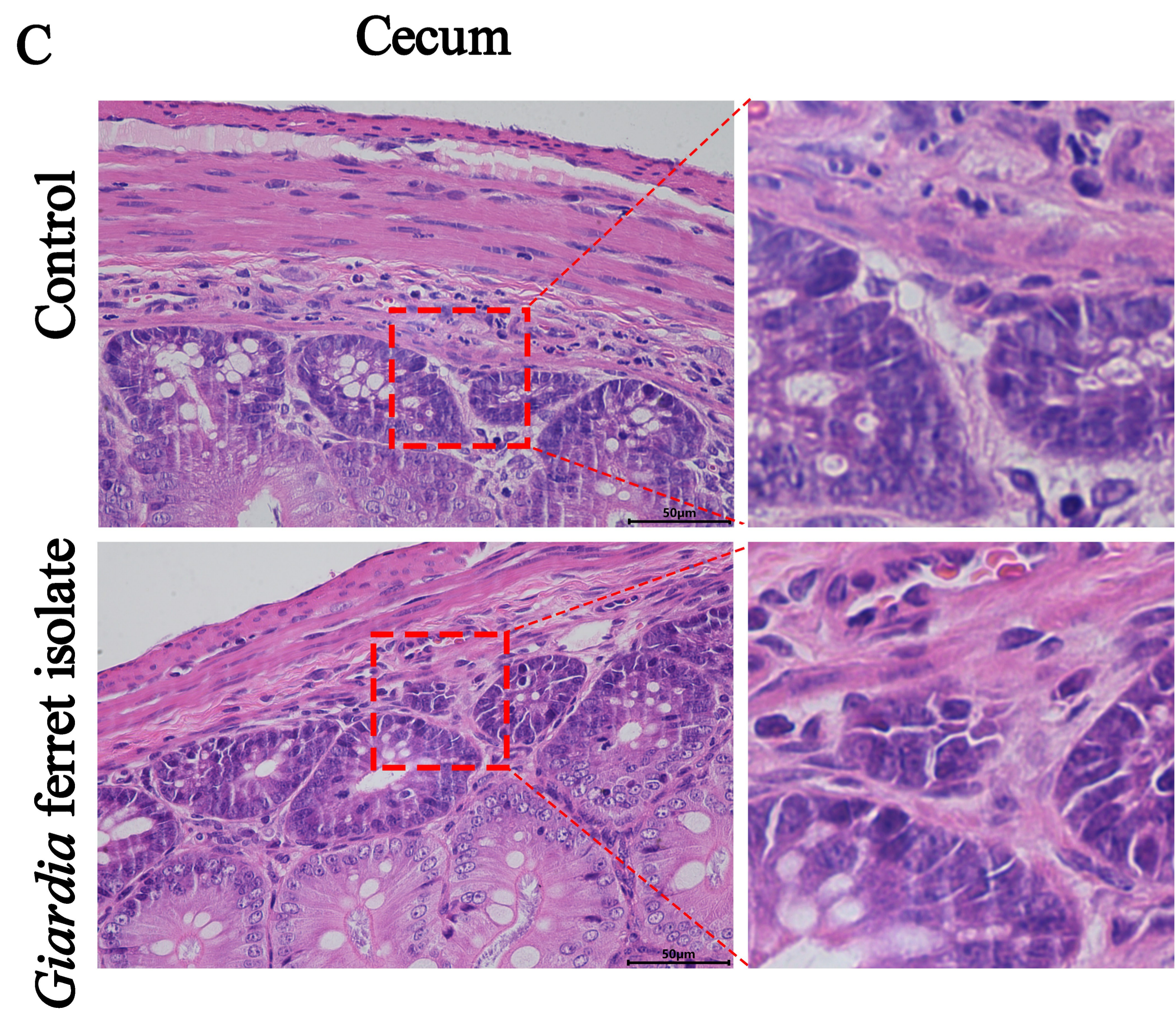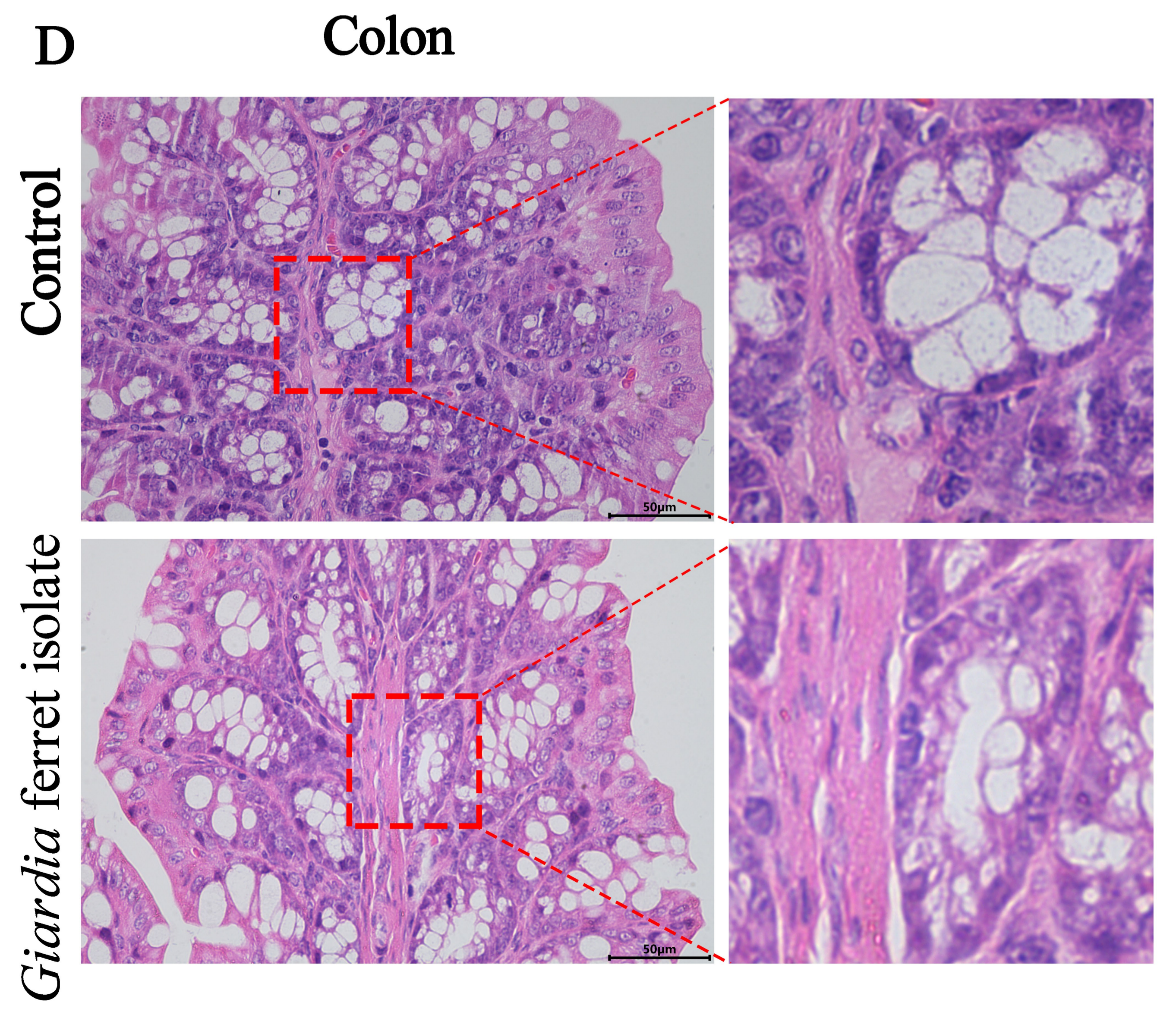

Supplement: Supporting Information 8 — Figure S4: Observation of pathological changes in intestinal tissue by HE staining. (A) Jejunum, (B) ileum, (C) cecum, and (D) colon. [file 3087035.f8.pdf]

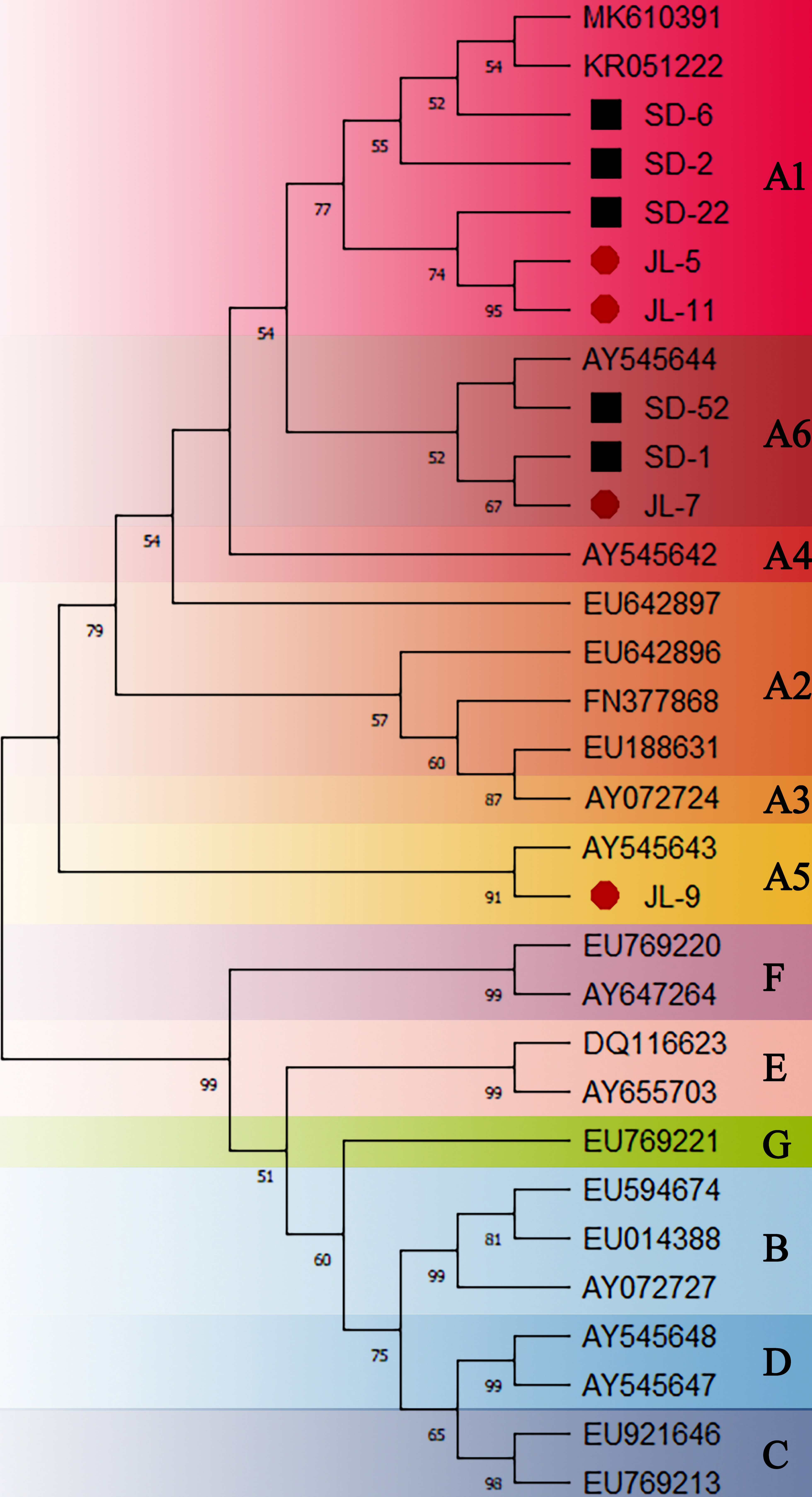

Supplement: Supporting Information 9 — Figure S5: The phylogenetic tree based on analysis of bg. [file 3087035.f9.pdf]

A

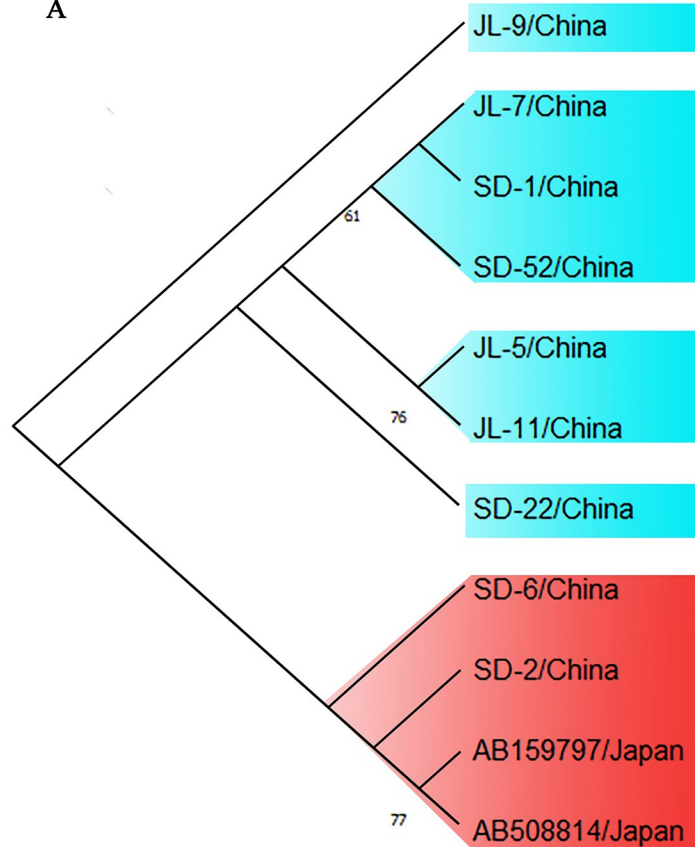

B

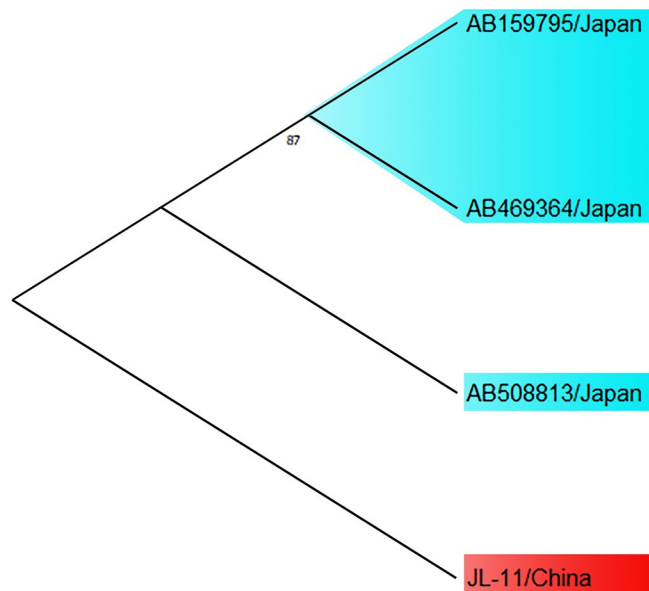

Supplement: Supporting Information 10 — Figure S6: Phylogenetic analysis of G. duodenalis sequences detected in this study and previously published from ferrets. (A) The phylogenetic tree based on analysis of bg. (B) The phylogenetic tree based on analysis of gdh. [file 3087035.f10.pdf]
