# Supplementary material for: First Characterization and Zoonotic Potential Evaluation of Giardia duodenalis in Ferrets in China
Source: Transbound Emerg Dis. 2025 May 29;2025:3087035. doi: 10.1155/tbed/3087035 (PMC12140828; doi:10.1155/tbed/3087035)

Giardia Assemblage A isolate WB 2019

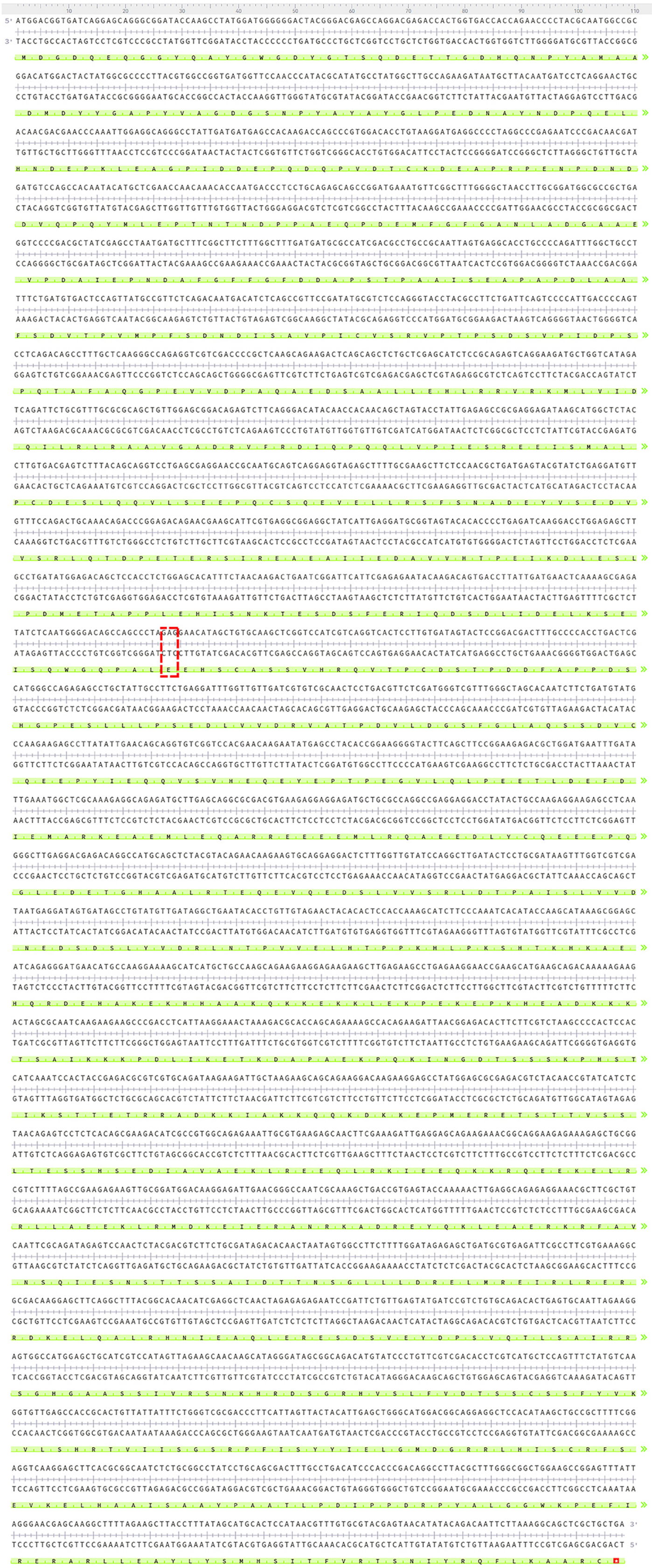

Giardia Assemblage A isolate Ferret

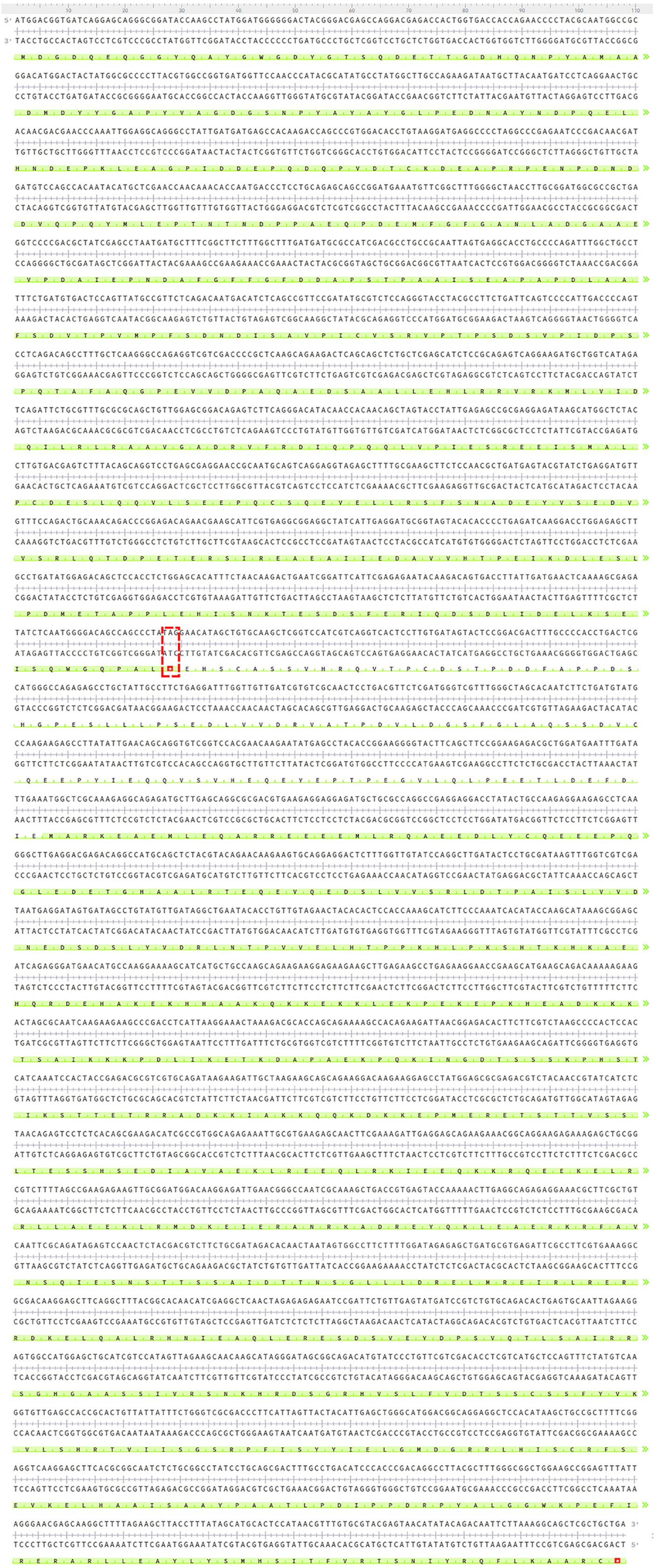

Supplement: Supporting Information 7 — Figure S3: SNP in PX domain-containing protein. [file 3087035.f7.pdf]
